# From Evidence-Based Research to Practice-Based Evidence: Disseminating a Web-Based Computer-Tailored Workplace Sitting Intervention through a Health Promotion Organisation

**DOI:** 10.3390/ijerph15051049

**Published:** 2018-05-22

**Authors:** Katrien De Cocker, Greet Cardon, Jason A. Bennie, Tracy Kolbe-Alexander, Femke De Meester, Corneel Vandelanotte

**Affiliations:** 1 Department of Movement and Sports Sciences, Ghent University, 9000 Ghent, Belgium; Greet.Cardon@ugent.be; 2 Institute of Resilient Regions, University of Southern Queensland, Springfield Central, QLD 4300, Australia; Jason.Bennie@usq.edu.au (J.A.B.); c.vandelanotte@cqu.edu.au (C.V.); 3 School of Health and Wellbeing, University of Southern Queensland, Ipswich, QLD 4305, Australia; Tracy.Kolbe-Alexander@usq.edu.au; 4 Flemish Institute Healthy Living, 1020 Brussels, Belgium; Femke.DeMeester@gezondleven.be; 5 Physical Activity Research Group, Appleton Institute, Central Queensland University, Rockhampton, QLD 4702, Australia

**Keywords:** sedentary behaviour, sitting time, worksite, computer-tailoring, employees, e-health, public health practice, dissemination, translation, implementation, RE-AIM

## Abstract

Prolonged sitting has been linked to adverse health outcomes; therefore, we developed and examined a web-based, computer-tailored workplace sitting intervention. As we had previously shown good effectiveness, the next stage was to conduct a dissemination study. This study reports on the dissemination efforts of a health promotion organisation, associated costs, reach achieved, and attributes of the website users. The organisation systematically registered all the time and resources invested to promote the intervention. Website usage statistics (reach) and descriptive statistics (website users’ attributes) were also assessed. Online strategies (promotion on their homepage; sending e-mails, newsletters, Twitter, Facebook and LinkedIn posts to professional partners) were the main dissemination methods. The total time investment was 25.6 h, which cost approximately 845 EUR in salaries. After sixteen months, 1599 adults had visited the website and 1500 (93.8%) completed the survey to receive personalized sitting advice. This sample was 38.3 ± 11.0 years, mainly female (76.9%), college/university educated (89.0%), highly sedentary (88.5% sat >8 h/day) and intending to change (93.0%) their sitting. Given the small time and money investment, these outcomes are positive and indicate the potential for wide-scale dissemination. However, more efforts are needed to reach men, non-college/university educated employees, and those not intending behavioural change.

## 1. Introduction

Recently, the increasing prevalence of sedentary behaviours (i.e., any waking behaviour characterized by an energy expenditure ≤1.5 metabolic equivalents (METs), while in a sitting, reclining or lying posture; [1]) in modern society [2], is an emerging public health concern. Current evidence links sedentary behaviours to several adverse physical and mental health outcomes [3,4], and high levels of sitting are common in many workplaces [5,6,7]. To combat the potential adverse health effects of prolonged sitting at work, an international group of experts [8] recommends that desk-based employees should gradually accumulate 2 h/day of standing and light activity (light walking) during working hours, and eventually accumulate 4 h/day of standing and light activity [8]. In addition, a core dual public health message promoting more physical activity and less sitting time is currently being encouraged by several governments (Australia, Belgium, etc.) [9,10]. Thus, workplace interventions aiming to replace and regularly break up sitting time with standing and walking should be promoted and implemented in order to improve public health and prevent chronic diseases at population level.

Recently the *Start to Stand* intervention was developed to change workplace sitting among office employees. This web-based, computer-tailored intervention [11], based on the Theory of Planned Behaviour (TPB) [12] and aspects of the Self-Regulation Theory (SRT) [13], provides its users with personalized feedback about their sitting time, as well as tips on how to change this after having completed an assessment questionnaire. We have shown that this web-based, computer-tailored intervention is feasible, acceptable and effective in reducing self-reported, workplace sitting among Flemish employees (mean change of −59 min/day after one month in the intervention group) [11,14].

To maximize public health impact, successful evidence-based health promotion interventions should be translated from a controlled research setting into the broader community under ‘real life’ conditions [15]. This is referred to as ‘dissemination’, defined as ‘a set of planned, systematic efforts designed to make a programme or innovation more widely available in practice’ [16,17]. Health promotion organisations are in key positions to disseminate existing evidence-based programs and deliver it to larger populations. However, there is little research evaluating such dissemination efforts [16,18]. Very often, dissemination is not a part of intervention studies that focus on effectiveness, and when it does happen, it is usually ad-hoc at the end of research projects and without evaluating how successful the dissemination really was [19]. The limited literature on this topic is also dominated by researcher-led dissemination efforts, and has had no focus on interventions targeting sitting [19]. Still, the World Health Organisation considers dissemination and implementation to be an important priority area for public health research [20]. Therefore, this study aimed to investigate: (i) how a health promotion organisation promoted the evidence-based, computer-tailored *Start to Stand* intervention to reduce workplace sitting, (ii) what investments were made (i.e., time and cost), and (iii) who was reached by the dissemination efforts.

## 2. Materials and Methods

### 2.1. Intervention Program ‘Start to Stand’

The evidence-based, computer-tailored website for reducing workplace sitting that was promoted by the local health promotion organisation is called ‘*Start to Stand*’. The development of this theory-driven intervention has been described in detail elsewhere [11,14,21]. In brief, users of *Start to Stand* register with the website by creating an account; they log onto the website, complete an initial assessment questionnaire and then immediately receive computer-tailored feedback (if they were employed and between 18–65 years at that time). A set of pre-defined decision rules selects feedback messages that are matched and tailored to the specific answers. The assessment questions obtain sitting time across different domains, job-related information, knowledge about sedentary behaviour, and constructs of the TPB [12] including attitudes, self-efficacy, social norm, and intention. The feedback messages contain details on the users’ sitting time and suggestions on how to interrupt (having short standing breaks) and reduce (replacing sitting by periods of standing) this. The combination of all feedback messages is referred to as ‘the advice’.

After receiving the advice, if interested, users are able to request up to 5 other non-committal specific sections. These additional sections are available immediately, but can be accessed at a later time. The focus of these additional sections is on standing breaks during working hours (Section 2a), replacing sitting by standing during working hours (Section 2b), sitting during commuting to work (Section 2c), sitting during (lunch) breaks at work (Section 2d), and on making an action plan to improve sitting behaviour (Section 2e) through SMART (Specific, Measurable, Attainable, Relevant and Time-bound) goals and implementation intentions [22,23].

### 2.2. Theoretical Framework for Dissemination Research

A commonly used framework in the evaluation of dissemination research is the RE-AIM framework [24,25]. This model determines the potential public health impact of behavioural interventions and consists of five dimensions, including ‘reach’, ‘efficacy/effectiveness’, ‘adoption’, ‘implementation’, and ‘maintenance’. *Reach* measures the number and characteristics of participants when compared to the target audience. *Efficacy or effectiveness* refers to the positive and negative consequences of a program on important outcomes under optimal conditions (efficacy) or in real-world situations (effectiveness). *Adoption* assesses delivery staff and setting variables. *Implementation* refers to the extent to which the program was implemented as intended in a real-world setting, i.e., intervention fidelity and resources (cost and time). Last, *maintenance* refers to continuing to deliver the program in the organisational and/or community setting over the long-term. Two dimensions operate at the individual level (*reach* and *efficacy*), while *adoption* and *implementation* are both organisational dimensions. *Maintenance* is a dimension operating at both the individual and organisational levels. As efficacy has been previously tested in a randomised controlled trial [14], this was not assessed in the present study. In addition, because of the cross-sectional nature of the study (no behavioural change was assessed among the website users), we did not examine the effectiveness of the intervention.

### 2.3. Data Collection and Data Analyses

In this case, the researchers did not lead the dissemination of the program. Therefore, the intervention outgrew the research setting, and a local health promotion organisation (the *Flemish Institute Healthy Living*) led the dissemination phase as the intervention became embedded in their system. The *Flemish Institute Healthy Living* is a governmental institute aiming to help citizens towards a healthy lifestyle, using evidence-based recommendations, education and interventions. The institute aims to help professionals and citizens to obtain healthy diets, to become physically active and less sedentary, to stop smoking and to improve mental health in the home and work setting. As the *Start to Stand* intervention is a fully developed, web-based program, not requiring printing or delivery costs, the *Flemish Institute Healthy Living* decided to include *Start to Stand* in its approach. As a result, the researchers asked the *Flemish Institute Healthy Living* staff member responsible for dissemination to systematically keep an inventory of their dissemination efforts (i.e., *adoption*, e.g., delivery staff and setting; and *implementation*, e.g., actions, cost and time investment) to promote the computer-tailored website among employees in Flanders (northern, Dutch-speaking part of Belgium; ~6,500,000 inhabitants in 2017; 2,768,000 working adults in 2016). This inventory was specifically developed for the present study. The components were selected by the researchers based on previous dissemination studies [24,25]; however, validity was not tested. Data were collected between October 2016 (making *Start to Stand* publicly available on the Internet) and the beginning of February 2018 (16 months after first launch). The terms and conditions of using the *Start to Stand* website mean that user information may be used for research purposes in such a manner that individual participants cannot be identified.

To report on the *reach* of the intervention, website usage descriptive statistics and data from Google Analytics were used. To describe the attributes of the website users, data collected during the initial assessment were used. This includes self-reported age, gender, education (low (no diploma, elementary school, secondary school) vs. high (high school, university)), average amount of time daily spent at work (hours-minutes/day), employment duration (number of years), height (cm) and weight (kg). Body mass index (BMI) was calculated with the following formula: weight/height^2^. The level of workplace sitting time was assessed using two items from the reliable and validated Workforce Sitting Questionnaire (WSQ) [26] in which users self-reported the average time spent sitting while being at work on work and non-workdays. The validated International Physical Activity Questionnaire (IPAQ) short version [27,28] was used to assess the number of days and duration of time spent in walking, moderate intensity physical activity and vigorous intensity physical activity in the last week. Based on the guidelines for data processing and analysis of the IPAQ [29], total scores for walking, moderate and vigorous intensity physical activities were computed (‘number of days’ × ‘duration of time’). 

Furthermore, five psychosocial factors were included in the assessment questionnaire (see [11,21] for details). Users’ *knowledge* about sedentary behaviour was asked. *Attitudes* towards changing sitting were measured using 6 items. *Self-efficacy* was measured by asking how certain employees were about changing their sitting (4 items). *Social support* as assessed by asking whether colleagues would support them when trying to change their sitting behaviour. Finally, employees’ *intention* to change sitting was asked. All these questions were based on previously validated questions to measure psychosocial correlates of physical activity [30] of which the wording was changed to reflect correlates of sitting [7]. All items were (re)coded into the same direction so that the highest scores were the most positive answers on each item. Cronbach’s α coefficients of internal consistency were calculated for attitudes (α = 0.68) and self-efficacy (α = 0.82) prior to computing the related items into one scale. To compare the users’ mean age, BMI, physical activity and sitting to that of the (total working) Flemish population [31], one-sample *t*-tests were used. To compare the gender distributions of the present sample to that of the total working Flemish population, a non-parametric X^2^-test was conducted. All statistical tests were done in IBM SPSS Statistics for Windows, version 24.0 (IBM Corporation, Armonk, NY, USA), and significance was set at *p* < 0.05.

## 3. Results

### 3.1. Dissemination Strategies of the Health Promotion Organisation (Adoption, Implementation and Maintenance)

Table 1 provides an overview of the dissemination methods used to promote this stand-alone, individual-based intervention over a period of sixteen months. Two teams of the *Flemish Institute Healthy Living* (team ‘physical activity and sedentary behaviour’ and team ‘healthy workplaces’) were involved in the dissemination; however, in practice, one staff member (trained health promoter with PhD degree in Health Sciences) coordinated the dissemination of *Start to Stand*. Mostly, online strategies (including the promotion of the *Start to Stand* website on the institute’s homepage; and sending e-mails, newsletters, Twitter, Facebook and LinkedIn posts to professional partners), and workshops and seminars were used (see Table 1).

**Figure 1:**
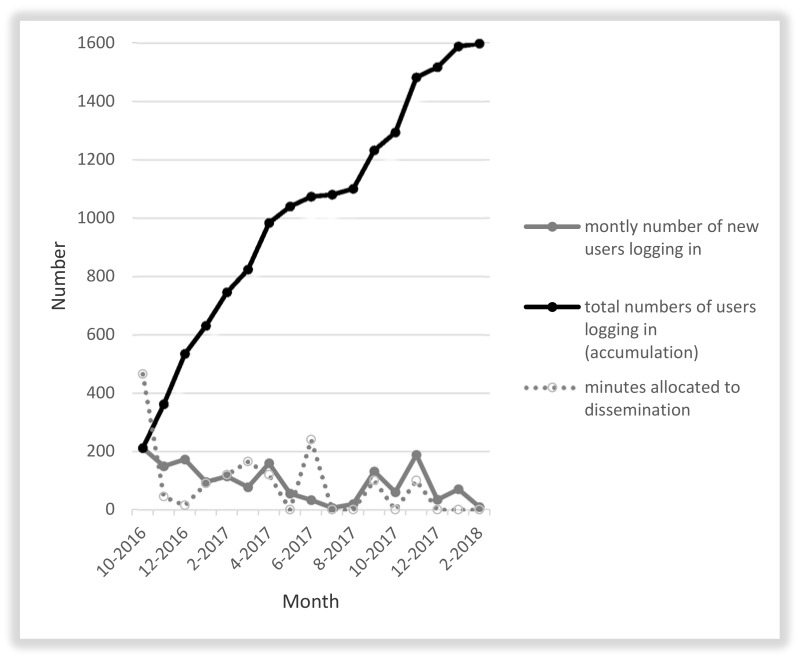
Number of minutes allocated to dissemination and number of visitors logging onto the website.

The total time investment was 25.6 h (summation of time spent on the several dissemination actions), and the total cost investment in salaries was approximately 845 EUR (25.6 × 33 EUR). One worked hour costs approximately 33 EUR, which includes the gross salary, bonuses, employer’s contributions, and fringe benefits. No other (print) costs were related to the dissemination. Figure 1 (dotted line) presents the number of minutes per month allocated to disseminating the intervention.

### 3.2. Users of the Evidence-Based Computer-Tailored Website (Reach and Maintenance)

From 12 October 2016 until 6 February 2018, we had 6906 unique visitors to the website. The majority (*n* = 4337, 60.6%) went directly to the website (using the URL directly or by clicking on a link for example within an email), 20% was done via website referral (mostly via the website of the team ‘healthy workplaces’ and the website of one of the partner organisations), 8.8% via social referral (91.3% via Facebook, 4.3% via LinkedIn and 3.8% via Twitter) and 8.1% via organic sources (e.g., search engines). The bounce percentage, i.e., percentage of visitors leaving the website after only viewing one page, was 51.3% (*n* = 3543). In total, 1599 visitors (*n* = 48.0% of unique visitors) created an account and logged onto the website. Figure 1 gives an overview of the number of individuals creating an account per month and the accumulative number of website users creating an account over time. Of the total sample logging onto the website (*n* = 1599), 11 (0.7%) did not further complete the assessment survey and stopped using the website after logging in. A total of 28 individuals (1.8%) was excluded because of their age (16 were <18 years, and 12 were >65 years). In addition, 60 individuals (3.8%) were excluded as they were not employed at that time. In total, 1500 users received the advice, which is estimated to be 0.05% of the working adult population in Flanders. On average, 1.02 min of time and 0.56 EUR were invested to reach one individual receiving the advice. The time invested aligned to a large extent with the number of employees reached each month (see Figure 1). Every month, about 100 individuals signed up to the website, except for the summer months, when many people are on holidays in Belgium (June–August). Despite the low efforts during recent months (December 2017, January 2018), new individuals were still visiting the website.

Demographic, work-related, health-related, and psychosocial characteristics of the sample completing the assessment survey and receiving the advice (*n* = 1500; 93.8%) are presented in Table 2. This sample was, on average, 38 years old, mainly female (76.8%), college/university educated (89.0%), highly sedentary (88.7% sat over 8 h/day) and sat on average 342 ± 121 min/day at work. People receiving the advice were significantly younger and consisted of more women than the total working population in Flanders (age: 43.4 years, t = −18.0, *p* < 0.001; gender: 46.6% female, X^2^ = 549.8, *p* < 0.001). The present sample had a significantly lower BMI compared to the general Flemish adult population (25.3 kg/m^2^, t = 12.0, *p* < 0.001) and reported less physical activity and more sitting compared to Flemish 15–85 year-olds (walking: 44.8 min/day, t = 48.5, *p* < 0.001; moderate physical activity: 33.5 min/day, t = 34.5, *p* < 0.001; vigorous physical activity: 18.0 min/day, t = 28.8, *p* < 0.001; sitting: 2506 min/week; 22% sat over 8 h/day, X^2^ = 3873.8, *p* < 0.001). 

The majority of the users was aware (84.7%) of the fact that sitting is related to adverse health outcomes, and 93.0% intended to change their sitting behaviours. Attitudes and self-efficacy towards changing sitting at work were, on average, relatively high. Only 10.5% reported that they had colleagues supporting them in changing their sitting at work (see Table 2).

A total of 521/1500 participants (34.7%) completed Section 2a (standing interruptions during working hours), 261/1500 (17.4%) completed Section 2b (prolonged standing during working hours), 185/1500 (12.3%) completed Section 2c (standing interruptions and prolonged standing during commuting), 171/1500 (11.4%) completed Section 2d (standing interruptions and prolonged standing during work breaks), and 224/1500 (14.9%) completed an action plan (Section 2e).

## 4. Discussion

We evaluated the dissemination of an existing and fully-developed web-based, computer-tailored intervention to reduce workplace sitting beyond its initial efficacy testing in a research context. A local health promotion organisation was responsible for spreading the intervention to its end users. To our knowledge, this is the first dissemination study of an individual program targeting workplace sitting using the RE-AIM framework. As a result, this study adds to the literature bridging the gap between research and practice in the public health promotion field. 

In this dissemination study, a single health promotion organisation was responsible for promoting the intervention. Two teams from the organisation were involved, but the dissemination was mainly coordinated by one employee of the organisation. This low staff involvement may explain why mostly online and social media strategies were used and why mostly professional partners (intermediary to reaching individual end users) were targeted by the health promotion organisation. The use of social media [15] and the engagement with stakeholders [32] have indeed been suggested as potential approaches for dissemination, as well as workshops and seminars [15], which were also used here. Dissemination approaches should be time-efficient [15], so the choice of these types of activities might have been appropriate in the given setting of limited staff involvement. In addition, time-efficient dissemination methods also keep the cost low, which is important, as local health promotion organisations often lack resources to disseminate theory-driven and evidence-based interventions. There is little information available on the costs of other dissemination projects, only six of the trials included in a review comparing dissemination studies using RE-AIM (*n* = 82) reported some data on monetary costs, ranging from 6.91 USD (~5.59 EUR) per person to 547 USD (~442.90 EUR) per person [24], which is much higher compared to the cost per person in the present study (0.56 EUR), suggesting that our dissemination was cost-efficient. A downside of using online social media is that the reach depends on how well connected the *Flemish Institute Health Living* is among social networks. Currently, the institute has about 1700 followers on Twitter and ~1400 on LinkedIn, the main channels used here. 

The dissemination reach of people actually getting the advice was approximately 0.05% of the Flemish working population. The review of Harden (2015) on dissemination studies found that the included behavioural interventions reached a median sample size of 320 participants (mean size = 4894 ± 28,256, range = 28–234,442) [24], so the present reach (*n* = 1500) is substantial. In addition, many more individuals came across the intervention and visited the website (*n* = 6906), but more than half of them did not create an account. This is not different from other research showing that a registration procedure can be a barrier for starting an intervention [33,34]. The current bounce rate of 51% can be considered average and is comparable to that of a Dutch Internet-delivered, computer-tailored lifestyle intervention (56%) implemented for use by the general public [35]. Further, it is possible that many more people would have been exposed to the messages of *Start to Stand*, but did not visit the website at all. 

The present sample of employees receiving the advice was mainly female, college/university educated, not overweight, rather low active, highly sedentary and intending to change this. Hence the intervention’s generalisability is potentially limited. This is, however, a common problem, as the review of behavioural interventions studies using RE-AIM showed that 46% of the studies that reported on representativeness (*n* = 17/82) found at least one significant difference between those that participated and the target population [24]. The fact that mostly women were reached (compared to the total working population in Flanders, the present study sample is more female) suggests that over-representation of women is not only a problem in research (predominantly more women are being recruited in health-related intervention studies [36]), but also in dissemination. Further, similar to our findings, studies in the review of Harden et al. (2015) showed that reached samples included more highly educated individuals [24]. Still, as the present intervention was developed to reduce workplace sitting, we reached the target population, i.e., sedentary employees, in term of behavioural attributes. Nevertheless, individuals not intending to change their sitting and employees unaware of the health risks related to too much sitting could not be targeted here.

Practical lessons learned from this study involve the need for easy, ready-to-use interventions, preferably internet-delivered, if the time and money resources of the promoter are limited, as this enhances dissemination. Further, practitioners are suggested to use online social media and to involve stakeholders. In addition, attempts should be made to reach those unaware of the health risks of sedentary behaviour, those not intending to change their sitting behaviours, male employees and lower-educated employees. Maybe these individuals can be better reached through other channels, for instance, sports clubs, television advertisements, or shopping centres. Further, more efforts are needed to encourage individuals to actually go to the website, log in, complete the survey and get the advice. Maybe the use of (inexpensive) incentives (e.g., drink bottles, key rings, or free health check-ups provided by the employer) can help here. In addition, as only small proportions of users (11–35%) completed one of the other non-committal sections, the additional value of these sections should be better promoted in the future.

The *Flemish Institute Health Living* is planning to embed the *Start to Stand* intervention in a larger multi-component program targeting workplace sedentary behaviour. Further research should examine whether these additional efforts, including a large communication campaign and the implementation of other socio-ecological intervention strategies, such as environmental and organisational changes in the workplace, can result in a higher reach of employees and a larger public health impact of the (office) workforce. Future studies could also assess the experiences and challenges of the health promoter and the target audience in order to provide a more detailed evaluation of, for example, barriers during implementation of the promoter, and communication preferences and barriers to register to the website among the end user.

A strength of this study is its added value to the literature, strengthening the global practice-based evidence, as there are few studies evaluating the dissemination of public health programs, especially ones focused solely on sitting interventions [19]. In addition, it should be noted that the present dissemination did not rely on trained research staff to deliver the intervention, but was embedded in existing practices of health promotion [17]. Another strength is that we reported on all but one dimension of the RE-AIM framework. The recent systematic review of behavioural interventions evaluated through the RE-AIM framework highlighted inconsistencies in the degree to which the RE-AIM dimensions were reported in its entirety and the inaccuracies in reporting indicators within each dimension [24]. It is, however, a limitation that the ‘real-world’ effectiveness of the intervention among the reached population is unknown. Nonetheless, based on the efficacy trial using self-reported data [14], one would assume the intervention could result in a reduction of sitting at work. Still, our findings are limited to the fact that we have no clear evidence of actual behavioural change. Further, the validity of the inventory is unknown, as only one staff member of the health promotion organisation completed details on actions and time investment. Finally, we were unable to determine the cost-effectiveness of the separate dissemination actions, so it is unknown which action is the most cost-effective.

## 5. Conclusions

We showed that with online and social media strategies of a health promotion organisation, a small proportion of the working adult population received our web-based, computer-tailored workplace sitting advice. Those reached were female, highly educated, normal-weight, highly sedentary, aware of the health risks of too much sitting, and intending to change their sitting. Given the small time and money investment, these outcomes are positive and indicate the potential for wide-scale dissemination of this web-based intervention, aiming to benefit more people and to foster policy and program development on a lasting basis [37]. However, more efforts are needed to reach male and non-college/university educated working adults, and employees unaware of the health risks of sedentary behaviour and not motivated yet to change their sitting.

## Figures and Tables

**Table 1:** Dissemination actions undertaken by the *Flemish Institute Healthy Living* to promote *Start to Stand*.

Timing	Dissemination Action“*Title of Message/Post*” (Length of Message/Post)	Potential Reach/*n* of Participants
12 Oct 2016	Newsletter to those subscribed to receive information on ‘healthy workplaces’:“*New Start to Stand tool helps employees to sit less*” (7 sentences and link to website)	1507 members
18 Oct 2016	News item on website of partner organisation: “*Start to stand*” (9 sentences and link to website)	5000 members
20 Oct 2016	Promoting *Start to Stand* during a training for partner organisations (15 min talk)	15 participants
21 Oct 2016	Twitter post from partner organisation (“*Tackling a sedentary lifestyle? @VIGeZine launches Start to Stand*”)	NA
28 Oct 2016	Referring to *Start to Stand* on homepage of theme ‘healthy workplaces’ (“*Start to stand! Find out more on how much you sit and how you can change it*”)	NA
3 Nov 2016	News item on website of theme ‘healthy workplaces’: “*New Start to Stand tool helps employees to reduce their sitting*” (6 sentences)	NA
14 Nov 2016	LinkedIn post from theme group ‘healthy workplaces’ (6 sentences)	714 members
13 Dec 2016	Promoting *Start to Stand* via email among network of local health promoters (1-page information letter)	15 partners
16 Jan 2017	Creating banner for email signature used by 5 staff members and 2 university staff members:“*Do you have a sitting job? And are you looking for tips to sit less?*” (2 sentences and link to website)	NA
1 Feb 2017	Referring to *Start to Stand* on website of theme ‘healthy workplaces’ (6 sentences and link to website)	NA
21 Feb 2017	Newsletter to members of partner organisations focussing on nutrition and physical activity:“*Sit less with Start to Stand*” (6 sentences and link to website)	1979 members
22 Feb 2017	Referring to *Start to Stand* on homepage of theme ‘nutrition and physical activity’ (6 sentences and link to website)	NA
22 Feb 2017	Referring to *Start to Stand* on 10,000 Steps be website (6 sentences and link to website)	NA
Feb 2017	Facebook post on 10,000 stappen.be Facebook page: “*New Start to Stand tool helps employees to reduce their sitting*” (6 sentences and link to website)	NA
7 Mar 2017	Promoting *Start to Stand* during a workshop of a partner organisation (10 min talk)	40 participants
17 Mar 2017	Newsletter to partner organisations focussing on socially responsible entrepreneurs (0.5-page letter)	NA
20 Mar 2017	Promoting *Start to Stand* during a presentation/seminar to prevention workers (10 min talk)	2 × 10 participants
22 Mar 2017	Promoting *Start to Stand* during a presentation/seminar regarding health and safety at work (10 min talk)	20 participants
30 Mar 2017	Twitter post from partner organisation (“*What about your sitting? Get up and do the Start to Stand test*”)	NA
5 Apr 2017	Twitter posts from 2 partner organisations (“*Reduce your sitting with Start to Stand*”)	NA
18 Apr 2017	Referring to *Start to Stand* on 2 partner websites (6 sentences and link to website)	NA
19 Apr 2017	Referring to *Start to Stand* on 3 partner websites (6 sentences and link to website)	NA
24 Apr 2017	Newsletter to those subscribed to receive information on ‘Healthy Living’: “*Flemings, start to stand!*” (8 sentences and link to website)	5493 members
22 Jun 2017	Promoting *Start to Stand* during a presentation/seminar regarding workplace physical activity targeting health promoters (40 min talk)	60 participants
29 Sep 2017	Promoting *Start to Stand* during a presentation/seminar of a partner organisation to health promoters (10 min talk)	35 participants
27 Nov 2017	Promoting *Start to Stand* during a presentation/seminar of a partner organisation (10 min talk)	25 participants

NA = not available.

**Table 2:** Characteristics of the website users completing the assessment survey.

Characteristics	Website Users (*n* = 1500)
*Demographic variables*	
Age: x ± SD years	38.3 ± 11.0
Gender: % (*n*) men	23.2 (348)
Education: % (*n*) high school/university	89.0 (1334)
*Work-related variables*	
Hours at work: x ± SD days	8.0 ± 1.1
Occupational status: % (*n*) white collar	98.1 (1472)
Employment duration: % (*n*) >5 years	54.3 (815)
*Health-related variables*	
BMI: x ± SD kg/m^2^	24.0 ± 4.1
Walking: x ± SD minutes/day	18.8 ± 20.4
Moderate-intensity PA: x ± SD minutes/day	16.3 ± 19.3
Vigorous-intensity PA: x ± SD minutes/day	8.9 ± 12.3
Total workday sitting: x ± SD minutes/week	3612.9 ± 960.9
Total non-workday sitting: x ± SD minutes/week	1124.8 ± 624.3
Sitting at work: x ± SD minutes/day	341.6 ± 120.6(~71% of time at work)
Sitting during transport: x ± SD minutes/day	81.1 ± 91.6
Sitting during TV viewing: x ± SD minutes/day	110.1 ± 65.0
Sitting during PC use: x ± SD minutes/day	77.5 ± 74.2
Other leisure time sitting: x ± SD minutes/day	99.7 ± 63.7
High level of sitting: % (*n*) reporting on average >8 h/day	88.5 (1328)
*Psychosocial variables related to sitting*	
Knowledge: % (*n*) agreeing that daily prolonged sitting can cause health problems	84.7 (1271)
Attitudes ^a^: x ± SD (range)	3.5 ± 0.8 (1.8–5.0)
Self-efficacy ^a^: x ± SD (range)	3.9 ± 0.6 (1.0–5.0)
Social support: % (*n*) agreeing that their colleagues encourage them to change sitting during working hours	10.5 (157)
Intention: % (*n*) intending to change sitting behaviours right away or in the next weeks	93.0 (1395)

SD = standard deviation; PA = physical activity; ^a^ mean score of 5-point scales ranging from ‘strongly disagree’ to ‘strongly agree’ (based on average of items).
